# Decreasing Trends in Road Traffic Mortality in Poland: A Twenty-Year Analysis

**DOI:** 10.3390/ijerph181910411

**Published:** 2021-10-03

**Authors:** Monika Burzyńska, Małgorzata Pikala

**Affiliations:** Department of Epidemiology and Biostatistics, The Chair of Social and Preventive Medicine of the Medical University of Lodz, Żeligowskiego 7/9, 90-752 Lodz, Poland; malgorzata.pikala@umed.lodz.pl

**Keywords:** road traffic, traffic accidents, mortality trends, death rates, epidemiology, Poland

## Abstract

The aim of the study was to assess mortality trends due to road traffic accidents in Poland between 1999 and 2018. The study material was a database including 7,582,319 death certificates of all inhabitants of Poland who died in the analyzed period (104,652 people died of transport accidents). Crude deaths rates (CDR), standardized death rates (SDR) and joinpoint models were used. Annual percentage change (APC) for each segment of broken lines and average annual percentage change (AAPC) for the whole study period were calculated. CDR decreased from 19.7 per 100,000 population in 1999 to 9.6 per 100,000 population in 2018; APC was −4.1% (*p* < 0.05) while SDR decreased from 20.9 to 10.9 per 100,000; APC was −4.1% (*p* < 0.05). Large differences in traffic accident-related mortality were observed between men and women. An analysis by gender and age shows that the decline in the number of deaths due to traffic accidents has been slowed down in the oldest age group, 65+, in both males and females. There is a need for in-depth analyses aimed at introducing effective preventive solutions in the field of road traffic safety in Poland. Legal regulations should particularly refer to the most endangered groups of road users.

## 1. Introduction

Mortality trends analysis is substantial in dealing with the consequences of diseases [1]. Demographic and socioeconomic development triggers changes in the pattern of morbidity and mortality, which is precisely explained by the theory of demographic transition, described in 1971 by Abdel R. Omran [2]. The problem of road accidents, the risk of which began to increase due to the development of motorization, triggered the need to extend this theory. Due to the fact that road accidents have become a serious epidemiological problem over several decades, a new category of mortality due to external causes of death and deaths related to pathological behavior, including those resulting from traffic accidents, was defined [3]. Traffic accidents represent one of the most important threats to public health. An analysis of international statistics on causes of death indicates that road accidents are one of the main causes of all injuries, which in turn are the third cause of deaths worldwide [4]. According to the World Health Organization (WHO), on average more than one million people die each year from road traffic accidents, and deaths resulting from multiple and multi-organ injuries sustained in those accidents constitute 2.2% of the global death toll. For every person killed in a road traffic accident, five people suffer permanent injuries, which results in a permanent change in the victims’ way of life. Research into the epidemiology and prevention of road accidents is particularly important due to the social impact of this phenomenon. Road accidents are also a serious problem for the state economy. They contribute to increased rates of disability, sickness absence, costs of treatment and rehabilitation, compensation and benefits, resulting in a loss of the Gross Domestic Product (GDP) each year. Globally, the annual cost of road traffic incidents is about 2% of GDP [5]. It is a particularly pressing issue faced by the transport sector, making road safety and mortality reduction key priorities. It has been receiving increased international attention, including through the launch of relevant initiatives aimed at stabilizing and then reducing the forecast level of road traffic fatalities. These include the Global Plan for the Decade of Action for Road Safety Collaboration, developed by the United Nations Road Safety Collaboration. Transport is very important for trade and the global economy. It facilitates access to services, helps develop the economy, reduces poverty and poses an important sustainable development issue. In 2015, road safety was expressly included in the 2030 Agenda for Sustainable Development [6]. Experts around the world are involved in activities for safety improvement, defining three approaches to road safety: the traditional approach (focuses more on human errors and road users), systemic approach (includes sustainable safety and safe systems) and vision zero (based on the principle that deaths and injuries caused by transport accidents can be prevented and may reach zero). The choice and implementation of road safety approach vary depending on the specificity of each country [7,8].

In 2019, there were 22,800 deaths due to road traffic accidents in the 27 European Union (UE) member states. The number of fatalities in Europe decreased by 23.0% between 2010 and 2019, but the pace of decline in the mortality rate in EU countries has decreased markedly recently; in the period 2018–2019, it was less than 2.0%. On average, 5 people per 100,000 inhabitants die annually in EU countries. The best situation has been observed in Sweden (2.2/100,000) and Ireland (2.9/100,000), while the worst situation has been noted in Romania (9.6/100,000), Bulgaria (8.9/100,000) and Poland (7.7/100,000).

Eight EU member states enjoyed the lowest rate ever in 2019, but the disparity between countries is very large. In countries with the worst epidemiological situation regarding road traffic accidents, the values of the detailed mortality rate due to this cause are four times higher than in those with the best epidemiological situation [9]. Targets set by the EU safety policy for 2021–2030 (a reduction in the number of fatalities and serious injuries by 50.0%) are grounds for global analyses aimed at introducing effective prevention and education solutions [10,11]. The identification of causes of insufficient road safety improvement must be preceded by an accurate epidemiological diagnosis.

In Poland, every fourth death being a result of external reasons occur because of traffic accidents. In 2019, 2909 persons were killed in road crashes (7.7 deaths per 100,000 inhabitants). A total of 1.0 road fatalities per 10,000 registered vehicles was recorded (compared to 4.5 in 2000). The analysis of fatalities by road type shows that in 2019 rural roads were the deadliest roads in Poland, accounting for 57% of all road deaths [12]. The main causes of crashes since 2004 are still speed inappropriateness for road conditions and failure to give way. The vast majority of crashes resulted in pedestrian casualties/fatalities and/or were a result of intoxicated road users. The Polish officials have implemented some methods aimed at increasing road safety in Poland. Those improvements evolve around modernizing the roads, penalizing for breaking a traffic law, novel regulations of technical condition of automobiles and educating road users [13]. A number of systematic actions were carried out, mainly the development and implementation of a sectoral National Road Safety Improvement Program—GAMBIT National Roads and the information campaign Roads of Trust [14].

Traffic accidents pose a great challenge for emergency services. It is essential to provide access to immediate health services due to a threat to health or life and to provide specialist rehabilitation for road accidents casualties in the post-accident phase. The modern medical emergency system should be designed to not only organize a network of ambulances or rescue helicopters but also to create appropriate emergency departments in which emergency medicine activities will be carried out. In Poland, where in addition to hospital emergency departments dedicated to patients with the most severe injuries were created, so-called trauma centers, dividing patients additionally into adult centers and children’s centers occurs. Currently functioning trauma centers in Poland are highly specialized health care facilities operating within the framework of the State Medical Rescue system and are an integral part of the health security system [15].

The number of deaths and injuries being a result of a road traffic accident in Poland is still substantial; what is more, Poland is one of the riskiest nations to drive among European countries. The social and economic impacts of a large number of road crashes and their consequences leading to death or disability are severe. The assessment of the epidemiological situation in this field is one of the most important points for the implementation of appropriate preventive measures.

The aim of this study was to assess trends in mortality due to road traffic accidents in Poland between 1999 and 2018. The results of the study may be essential to allocate appropriate methods and resources to promote interventions that are effective at achieving outcomes in the context of road safety, mainly in high-risk groups.

## 2. Materials and Methods

The study was conducted according to the guidelines of the Declaration of Helsinki, and approved by the Bioethics Committee of the Medical University of Lodz on 22 May 2012 No. RNN/422/12/KB. 

The study material was a database including 7,582,319 death certificates of all inhabitants of Poland who died in the period 1999–2018. Of this number, 104,652 people died from transport accidents (according to the International Statistical Classification of Diseases and Health Related Problems—Tenth Revision—ICD-10, coded as V01-V99, Y85). The data were provided by the Department of Information of the Polish Central Statistical Office.

The authors calculated crude deaths rates (CDR) and standardized death rates (SDR).

$$CDR=\frac{k}{p}*100,000$$

where *k* is the number of transport accidents deaths; *p* is the population size.

$$SDR=\frac{\sum_{i=1}^{N}\frac{ki}{pi}wi}{\sum_{i=1}^{N}wi}$$

where *k_i_* is the number of transport accidents deaths in this i-age group, *p_i_* is the population size of this i-age group, *w_i_* is the weight assigned to this i-age group, resulting from the distribution of the standard population, and *N* is the number of the age groups.

The standardization procedure was performed with the use of the direct method, in compliance with the European Standard Population, updated in 2012 [16]. Revised European Standard Population is the unweighted average of the individual populations of EU-27 plus European Free Trade Association (EFTA) countries in each five-year age band (with the exception of under 5 and the highest band, 85+).

The analysis of time trends has been carried out with joinpoint models and the Joinpoint Regression program, a statistical software package developed by the U.S. National Cancer Institute for the Surveillance, Epidemiology and End Results Program (Maryland, United States) [17].

The joinpoint regression model is an advanced version of linear regression *y* = bx + a, where: b is the slope coefficient, a is the *y*-intercept. *y* = ln(z), z is a measure evaluated in the study (CDR, SDR) and x is the calendar year. Time trends were determined with the use of segments joining in joinpoints, where trend values significantly changed. To confirm whether the changes were statistically significant, the Monte Carlo Permutation method was applied.

In addition, the authors also calculated the Annual Percentage Change (APC) for each segment of broken lines and the Average Annual Percentage Change (AAPC) for the whole study period with corresponding 95% confidence intervals (CI).

Annual Percent Change is one way to characterize trends in death rates over time, and it was calculated according to the following formula:

$$APC=100*(exp^{b}-1)$$

where *b* is the slope coefficient.

With this approach, the death rates are assumed to change at a constant percentage of the rate of the previous year. For example, if the APC is 1%, and the rate is 50 per 100,000 in 2000, the rate is 50 × 1.01 = 50.5 in 2001 and 50.5 × 1.01 = 51.005 in 2002. Rates that change at a constant percentage every year change linearly on a log scale.

The Average Annual Percent Change (AAPC) is a summary measure of the trend over a pre-specified fixed interval. It allows us to use a single number to describe the average APCs over a period of multiple years. It is valid even if the joinpoint model indicates that there were changes in trends during those years. It is computed as a weighted average of the APCs from the joinpoint model, with the weights equal to the length of the APC interval [18].

$$AAPC=\{exp(\frac{\sum wibi}{\sum wi})-1\}\times 100$$

where *b_i_* is the slope coefficient for each segment in the desired range of years and *w_i_* is the length of each segment in the range of years.

## 3. Results

The number of deaths due to transport accidents in 1999 was 7537, which accounted for 2.0% of all deaths of Polish residents (Table 1).

**Figure 1:**
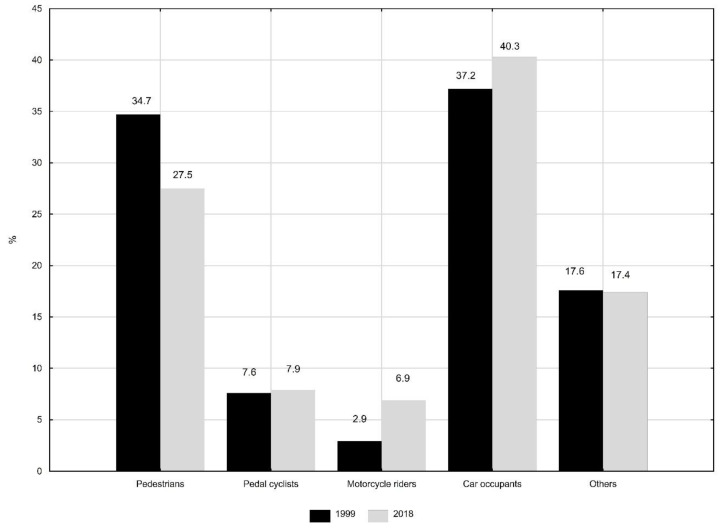
Structure of deaths due to traffic accidents by causes (%). Source: own calculations.

After 20 years, in 2018, the number of deaths decreased to less than half and was 3672 (0.9% of all deaths of Polish residents). Car occupant injuries made up the largest group of causes of death due to transport accidents. In 1999, deaths due to this group of causes among all transport accidents constituted 37.2%, in 2018, the value increased to 40.3% (Figure 1).

The second most common group included deaths due to pedestrians injured in transport accidents. However, the percentage value decreased over the analyzed 20 years from 34.7% to 27.5%. The mortality related to motorbike rider injuries increased from 2.9% in 1999 to 6.9% in 2018. The proportion of deaths due to pedal cyclist injuries remained similar (7.6% and 7.9%, respectively).

The crude death rate (CDR) due to traffic accidents decreased from 19.7 per 100,000 population in 1999 to 9.6 per 100,000 population in 2018 (Table 1). The Annual Percentage Change (APC) was −4.1% (*p* < 0.05) (Table 2).

**Figure 2:**
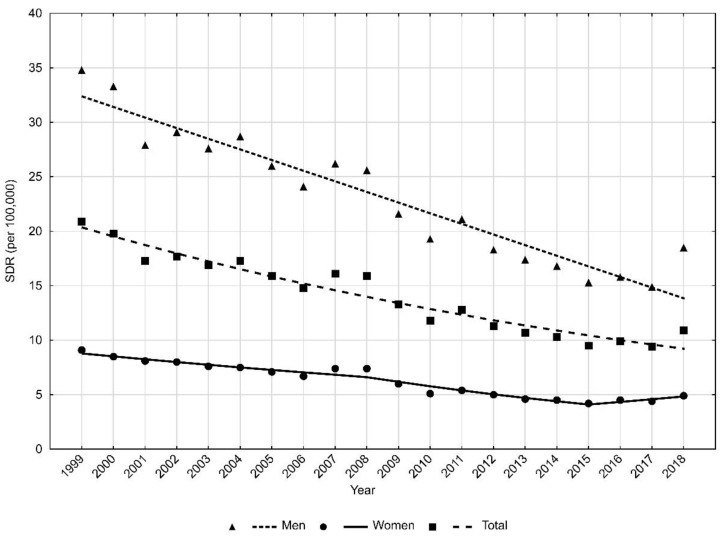
Time trends of standardized death rates (SDR) due to road traffic injuries in 1999–2018 in Poland. Source: own calculations.

The standardized death rate (SDR) for 1999 was 20.9, but in 2018, its value decreased to 10.9 per 100,000 population. The APC was −4.1% (*p* < 0.05) (Figure 2).

Large differences in traffic accident-related mortality were observed between men and women. In the male group, CDR in 1999 was 31.7 per 100,000 (Table 1). Over 20 years, CDRs decreased at an annual rate of −4.2% (*p* < 0.05) (Table 2) to 15.1 per 100,000 in 2018. Standardized death rates in the male group decreased from 34.8 in 1999 to 18.5 in 2018 (APC = −4.2%, *p* < 0.05) (Figure 2).

In the female group, CDRs values decreased from 8.4 in 1999 to 4.4 in 2018. The rate of decline was statistically significant, and it changed twice in the 20-year analyzed period. Between 1999 and 2008, the APC was −2.2% (*p* < 0.05). Between 2008 and 2013, the rate of decline was faster, i.e., −8.1% (*p* < 0.05). After 2013, the rate of decline was only −0.1% (*p* > 0.05). The Average Annual Percentage Change (AAPC) for the whole period of 1999–2018 was −3.3% (*p* < 0.05).

SDRs due to traffic accidents in the female group were 9.1 per 100,000 in 1999 and 4.9 in 2018. The rate of decline changed twice. Between 1999 and 2008, it was −3.1% (*p* < 0.05); between 2008 and 2015, the decline accelerated to −6.6% (*p* < 0.05). Between 2015 and 2018, the trend reversed, and SDRs increased at an average annual rate of 5.6% (*p* > 0.05). The AAPC for the entire 20-year period of 1999–2018 was −3.1% (*p* < 0.05).

Huge age-related differences in traffic accident mortality were also observed. In the male group, the highest SDR values were noted in subjects aged 45–64 years (Table 3).

**Figure 3:**
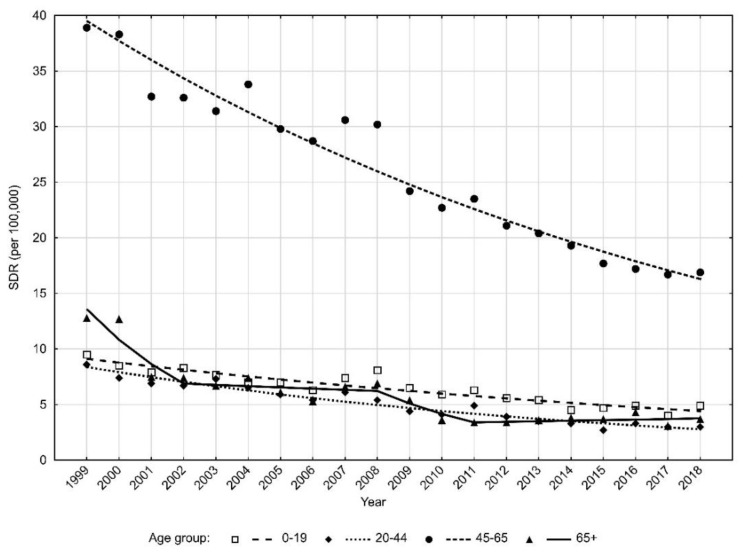
Time trends of standardized death rates (SDR) due to road traffic injuries in men according to age groups in 1999–2018 in Poland. Source: own calculations.

The SDR value in this age group was 38.9 in 1999 and 16.9 in 2018 (APC = −4.6%, *p* < 0.05) (Table 4). Significantly lower index values were observed in the other age groups (Figure 3).

In the oldest age group, i.e., above 65 years, SDRs decreased from 12.8 to 3.7 (AAPC = −6.5, *p* > 0.05). Trends of change in this age group changed three times, and the most rapid and statistically significant decline occurred between 1999 and 2002 (APC = −20.3%, *p* < 0.05).

For men aged 0–19 years, SDRs decreased from 9.5 in 1999 to 4.9 in 2018 (APC = −3.7%, *p* < 0.05). The lowest SDRs due to traffic accidents in the male group were noted for the age 20–44 years. In 1999, the SDR was 8.6, whereas in 2018, it was 3.0 (APC = −5.6%, *p* < 0.05).

**Figure 4:**
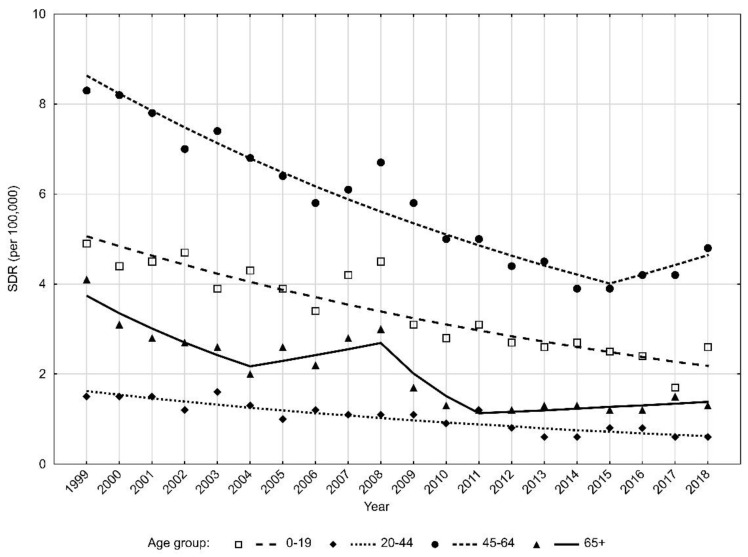
Time trends of standardized death rates (SDR) due to road traffic injuries in women according to age groups in 1999–2018 in Poland. Source: own calculations.

Slightly smaller differences between age groups were observed in the female group rather than in the male group (Figure 4).

The highest SDR values were noted for women aged 45–64 years (Table 3). Their value decreased from 8.3 in 1999 to 4.8 in 2018. This trend changed in 2015. Between 1999 and 2015, SDRs decreased at a rate of −4.7% (*p* < 0.05) but between 2015 and 2018, SDRs began to increase at a rate of 5.0% (*p* > 0.05) (Table 4).

In the oldest group of women (65+), SDRs decreased from 4.1 in 1999 to 1.3 in 2018 (AAPC = −5.1%, *p* > 0.05) and the trend changed three times. Only the decrease observed between 1999 and 2004 was statistically significant (APC = −10.3%, *p* < 0.05). In the 2011–2018 period, SDR values increased at a rate of 2.9% (*p* > 0.05).

In the group of females aged 0–19 years, SDRs values decreased from 4.9 in 1999 to 2.6 in 2018 (APC = −4.3%, *p* < 0.05). The lowest SDR values were observed in women aged 20–44 years, and they were the following: 1.5 in the year 1999 and 0.6 in the year 2018 (APC = −5.0%, *p* < 0.05).

## 4. Discussion

The Global Status Report on Road Safety published by the World Health Organization in 2018 points out the effectiveness of actions taken in most countries in recent years that aim at improving road safety [19]. One of the key tasks carried out in this area is the educational activities both in the general population and among road safety specialists. The modernization of road infrastructure, elimination of vehicles in a poor technical condition and posing a traffic threat, the introduction of stricter regulations and unavoidable punishment for road traffic offenses, as well as first aid education, brought substantial effects. A large role in this regard is also attributed to an improvement of the quality and availability of professional medical assistance at the scene of an accident, as well as the application of more effective diagnostic, therapeutic and rehabilitation methods [20]. Between 1990 and 2017, despite the global downward trend in mortality due to transport accidents, many countries, such as Paraguay, Pakistan, Mongolia and North Korea, experienced negative trends in age-standardized rates [21]. This fact may be explained, among others, by results of studies conducted in the United States, indicating that accident victims who sustained moderate or serious injuries and who go to trauma centers demonstrate a 25% lower risk of death than those who do not go to professional trauma centers, which in turn happens much more often in low-income countries [22]. The WHO reports that the mortality rate due to motorbike accidents is also increasing globally (from 14.9% to 18.2% between 1990 and 2017) [23]. This observation was also confirmed in our study.

However, in most countries of the world, including the European region, a decrease in mortality rates due to transport accidents has been observed over the last two decades. On average, a 29.0% decrease in age-standardized mortality rates due to this cause was noted worldwide between 1990 and 2017 (15.8 per 100,000 in 2017). In European Union countries, the rate decreased on average by 23.0% between 2010 and 2019. The largest decrease was observed in Greece, Spain and Portugal [24]. As shown in this study, a similar trend was also observed in Poland. The standardized death rate (SDR) in the year 1999 was 20.9, while in 2018, it was 10.9 per 100,000 inhabitants, and the average rate of decline in the analyzed period was 4.1%.

The behavior of road users is one of the most important determinants of road safety. Inappropriate speed, in particular, is one of the main causes of road crashes. In Poland, between 2001 and 2017, the number of fatal crashes involving speeding has decreased by 54%. However, speed remains one of the main causes of crashes, and it is a contributing factor in 35% of fatalities, based on data from 2019. Speed enforcement efforts are constantly, increasing and new regulations regarding automatic speed enforcement are being introduced into Polish law. Road accidents on national roads account for 19% of all accidents, with fatalities representing as much as 33% of all fatalities. The rate has decreased by 55% since 2001. Fatal accidents usually happen in the absence of daylight or due to fatigue or drivers nodding off. In 2017, as many as 45% of all fatalities died in these types of accidents. Between 2001 and 2017, the number of people killed in road accidents caused by drivers during the night dropped by 42%. In the late 1990s, alcohol was one of the main road safety problems in Poland. Over the last 10 years, the number of crashes caused by drivers under the influence of alcohol has decreased by 33%. According to police data, 9.1% of traffic fatalities were alcohol-related in 2019. In this respect, Poland tops the list as a country with the lowest share of fatal drinking and driving accidents. This is a result of a number of radical and systematic efforts by traffic enforcement services, education campaigns and by a new culture of alcohol consumption in Poland [12,25].

Since 1991, when the peak in the number of fatalities was reached, some legislation and policies have been implemented in Poland. Those included compulsory seat belt wearing for all car occupants (1991), a demerit point system (1998), the compulsory use of child restraints (1998), the appointment of the National Road Safety Council (2002), 50 km/h speed limit in built-up areas (2004), daytime running lights (2007), speed enforcement (including automatic speed enforcement) (2011), implementation of the EU directive on road safety management (2012), changes in the driver education system (2013), increased severity of penalties for speeding (2015), severe penalties for drunk driving (2015), development of the National Road Safety Programme 2013–2020, pedestrians’ priorities and an all-day speed limit in built-up areas (2021) [12].

It is noteworthy that men are more likely to die from road accidents [26]. Our own study showed that despite favorable trends in both groups, mortality rates in the male group were almost four times higher than in the female group, both at baseline and in the final year of the analysis and this trend applied equally to raw and standardized rates. Data from the Motor Transport Institute reveal that women cause one accident per 6.7 million covered kilometers. With regards to men, the distance is 4.7 million kilometers [27]. A similar pattern is observed in EU countries, where on average, 76% of fatalities in road traffic accidents are males, and no changes in this respect have been observed since 2010 [28]. The reasons for this phenomenon are most often attributed to the fact that women drive more carefully, are more focused on driving and more often observe traffic regulations. Road safety is not only determined by the driving technique, which is often better in men, but also by risk awareness and responsibility, which are more typical of women rather than men [29]. However, in recent years, women have become more professional and social [30], which results in their increased involvement in road accidents. The research results indicate that women are driving more and more recklessly. This trend was confirmed in this study, too. Since 2015, the mortality rate in the group of women aged 45 years and older has been dramatically increasing.

The author’s own study also showed differences in the pattern of mortality due to traffic accidents by age groups. The highest rates were recorded in the age group 45–64 years, both for men and women. However, the trends were downward, except for the oldest age groups, which is also confirmed by data of the European Commission for member states. The statistics show that between 2010 and 2018, the mortality rate due to transport accidents in the group of people over 65 years of age increased from 28.0% to 38.0% in urban areas [31]. Similar results were obtained in studies conducted in the Netherlands, where the most unfavorable trends in the age-standardized mortality rate due to transport accidents were recorded just in the age group 64+ [32]. Most studies analyzing this aspect show that the oldest drivers demonstrate a much higher risk of accidents, including fatal ones than middle-aged drivers. Furthermore, there are also differences in subgroups. It has been confirmed that drivers aged 70–74 years are burdened with a lower accident risk compared with drivers aged 75–79 years. However, drivers aged 80 years and over are at the highest risk of causing a fatal accident [33,34]. Data from the European Commission indicate that the type of accident depends on the age of the victim. Pedestrian mortality most often concerns children under 10 years of age and older people (65+), and in both groups, it accounts for about 40.0% of all deaths in this class of causes (twice as much as in other age groups). A similar pattern is observed in the group of people who die in a cycling accident [35]. This pattern is confirmed in a study conducted in Germany, which showed that both pedestrians and cyclists aged 65 and older are significantly more likely to die in a collision with a vehicle than other age groups [21]. Older people are less physically and mentally capable of coping in heavy traffic conditions. They often demonstrate uncertainty and anxious reactions, which, combined with deteriorated motor coordination, as typical for the elderly, increase the risk of accidents [36]. Age-related causes of accidents include health conditions, increased reaction time, visual impairment and inability to properly assess the distance [37]. The issue of young drivers being at high risk of dying in a traffic accident is also important. While referring to results of a study conducted in Poland, we can note that the mortality rate from this cause was not the highest among people aged 20–44 years, but it should be pointed out that deaths in this group accounted for 58.0% of all deaths in this class in 1990, while in 2018, deaths made up almost half (49.6%) of the total number of deaths. This pattern was similar to the one observed in other countries, but the rate of decline was different. In contrast, in Germany in 2017, deaths due to transport accidents in the age group 15–49 years accounted for 41.6% of the total number of deaths from this cause (60.5% in 1990). In the same year, in France, the value was 50.1% (51.3% in 1990), and in Italy 41.7% (52.9% in 1990) [38]. In Poland, however, the values of standardized detailed mortality rates due to this cause were higher than in most EU countries. On the basis of studies on transport accident-related deaths in the group of novice drivers, it can be concluded that the mortality risk due to this cause is strongly related to the age of the victims. According to WHO data, every third accident (37.31%) occurs in people aged 25–49 years [39]. Data from the Polish police and studies conducted by insurance companies show that drivers aged 18–24 cause the greatest number of accidents. In 2019, they contributed to 18.5% of all accidents caused by drivers. A failure to adapt their speed to road conditions was the major cause of accidents. It is particularly worrying that these accidents were associated with the highest mortality rate, i.e., 53.2% [40]. Thus, this problem is very important in terms of reducing mortality from this cause in young people.

Traffic accidents are among the major global health problems. In 2017, they accounted for 57,638,366 years of life lost (YLLs), 10,159,667 years of life with disability (YLDs) and 67,798,033 years of life lost due to disability (DALYs) (WHO, 2018). Trends in mortality due to traffic accidents can be shaped by both external and behavioral factors, especially with regard to age and gender [41]. Despite favorable trends, the rate of improvement in transport accident mortality has declined in recent years, and the risk of death due to this cause is still high.

We are aware of the limitations of the present study. It may be limited by the fact that there is no information about the cause of the fatal accident. A set of detailed victim characteristics per road traffic crash cause is not available; therefore, it is impossible to provide statistical correlations or predictions between different aspects of fatal accidents. We believe, however, that the present twenty-year analysis of data from 7,582,319 death certificates, including 104,562 deaths due to transport accidents, contributes to the literature and allows international comparisons of our data.

## 5. Conclusions

The number of deaths due to traffic accidents has been steadily decreasing between 1999 and 2018. A detailed analysis by gender and age shows that this decline has, however, been slowed down in the oldest age group, 65+, in both males and females. Young drivers still constitute a high-risk group. Their deaths from traffic accidents account for half of the total number of deaths in this class. The number of pedestrian deaths is decreasing; however, it is still high. On the other hand, the number of deaths of motorbike users is increasing.

There is a need for in-depth analyses aimed at introducing effective preventive and educational solutions in the field of road traffic safety in Poland. Legal regulations should particularly refer to the most endangered groups of road users.

## Figures and Tables

**Table 1:** Number of deaths and values of CDR (per 100,000) and SDR (per 100,000) due to road traffic injuries in Poland, 1999–2018.

Year	Men			Women			Total		
	Number of Deaths	CDR	SDR	Number of Deaths	CDR	SDR	Number of Deaths	CDR	SDR
1999	5873	31.7	34.8	1664	8.4	9.1	7537	19.7	20.9
2000	5556	30.0	33.3	1557	7.9	8.5	7113	18.6	19.8
2001	4797	25.9	27.9	1507	7.6	8.1	6304	16.5	17.3
2002	5006	27.0	29.1	1498	7.6	8.0	6504	17.0	17.7
2003	4815	26.0	27.6	1426	7.2	7.6	6241	16.3	16.9
2004	4977	26.9	28.7	1429	7.3	7.5	6406	16.8	17.3
2005	4609	25.0	26.0	1354	6.9	7.1	5963	15.6	15.9
2006	4302	23.3	24.1	1272	6.5	6.7	5574	14.6	14.8
2007	4655	25.3	26.2	1422	7.2	7.4	6077	15.9	16.1
2008	4607	25.0	25.6	1429	7.2	7.4	6036	15.8	15.9
2009	3876	21.0	21.6	1176	6.0	6.0	5052	13.2	13.3
2010	3544	19.0	19.3	999	5.0	5.1	4543	11.8	11.8
2011	3820	20.5	21.1	1030	5.2	5.4	4850	12.6	12.8
2012	3288	17.6	18.3	969	4.9	5.0	4257	11.0	11.3
2013	3132	16.8	17.4	887	4.5	4.6	4019	10.4	10.7
2014	2968	15.9	16.8	872	4.4	4.5	3840	10.0	10.3
2015	2725	14.7	15.3	802	4.0	4.2	3527	9.2	9.5
2016	2785	15.0	15.8	871	4.4	4.5	3656	9.5	9.9
2017	2632	14.2	14.9	848	4.3	4.4	3480	9.1	9.4
2018	2806	15.1	18.5	866	4.4	4.9	3672	9.6	10.9

**Table 2:** Time trends of CDR and SDR due to road traffic injuries in Poland in 1999–2018—joinpoint regression analysis.

	Number of Joinpoints	Years	APC (95% CI)	AAPC (95% CI)
Total				
CDR	0	1999−2018	−4.1 * (−4,6; −3.6)	
SDR	0	1999−2018	−4.1 * (−4.6; −3.5)	
Men				
CDR	0	1999−2018	−4.2 * (−4.7; −3.6)	
SDR	0	1999−2018	−4.2 * (−4.8; −3.5)	
Women				
CDR	2	1999−2008	−2.2 * (−3.5; −0.9)	
		2008−2013	−8.1 * (−12.2; −3.8)	−3.3 * (−4.6; −1.9)
		2013−2018	−0.1 (−3.3; 3.1)	
SDR	2	1999−2008	−3.1 * (−4.3; −1.9)	
		2008−2015	−6.6 * (−8.7; −4.4)	−3.1 * (−4.4; −1.8)
		2015−2018	5.6 (−1.2; 13.0)	

* *p* < 0.05.

**Table 3:** Standardized death rates due to road traffic injured by sex in 1999−2018 in Poland according to age groups.

Year	0–19		20–44		45–64		65+	
	Men	Women	Men	Women	Men	Women	Men	Women
1999	9.5	4.9	8.6	1.5	38.9	8.3	12.8	4.1
2000	8.5	4.4	7.4	1.5	38.3	8.2	12.7	3.1
2001	7.9	4.5	6.9	1.5	32.7	7.8	7.5	2.8
2002	8.3	4.7	6.7	1.2	32.6	7.0	7.4	2.7
2003	7.7	3.9	7.3	1.6	31.4	7.4	6.7	2.6
2004	7.0	4.3	6.5	1.3	33.8	6.8	7.4	2.0
2005	7.0	3.9	5.9	1.0	29.8	6.4	6.1	2.6
2006	6.3	3.4	5.4	1.2	28.7	5.8	5.3	2.2
2007	7.4	4.2	6.1	1.1	30.6	6.1	6.6	2.8
2008	8.1	4.5	5.4	1.1	30.2	6.7	6.9	3.0
2009	6.5	3.1	4.4	1.1	24.2	5.8	5.4	1.7
2010	5.9	2.8	4.1	0.9	22.7	5.0	3.6	1.3
2011	6.3	3.1	4.9	1.2	23.5	5.0	3.4	1.2
2012	5.6	2.7	3.9	0.8	21.1	4.4	3.4	1.2
2013	5.4	2.6	3.5	0.6	20.4	4.5	3.6	1.3
2014	4.5	2.7	3.3	0.6	19.3	3.9	3.8	1.3
2015	4.7	2.5	2.7	0.8	17.7	3.9	3.7	1.2
2016	4.9	2.4	3.3	0.8	17.2	4.2	4.3	1.2
2017	4.0	1.7	3.0	0.6	16.7	4.2	3.1	1.5
2018	4.9	2.6	3.0	0.6	16.9	4.8	3.7	1.3

**Table 4:** Time trends of SDR due to road traffic injuries in 1999–2018 in Poland by sex and age groups—joinpoint regression analysis.

Age Group	Number of Joinpoints	Years	APC (95% CI)	AAPC (95% CI)
**Men**				
0–19	0	1999–2018	−3.7 * (−4.4; −3.0)	
20–44	0	1999–2018	−5.6 * (−6.3; −5.0)	
45–64	0	1999–2018	−4.6 * (−5.0; −4.1)	
65+	3	1999–2002	−20.3 * (−35.3; −1.7)	−6.5 (−13.0; 0.4)
		2002–2008	−1.7 (−10.5; 8.0)	
		2008–2011	−18.2 (−46.2; 24.3)	
		2011–2018	1.4 (−4.1; 7.2)	
**Women**				
0–19	0	1999–2018	−4.3 * (−5.3; −3.4)	
20–44	0	1999–2018	−5.0 * (−6.1; −3.8)	
45–64	1	1999–2015	−4.7 * (−5.4; 4.0)	−3.2 * (−4.7; −1.7)
		2015–2018	5.0 (−4.8; 15.8)	
65+	3	1999–2004	−10.3 * (−16.3; −3.9)	−5.1 (−10.2; 0.2)
		2004–2008	5.5 (−9.6; 23.1)	
		2008–2011	−25.2 (−45.1; 1.8)	
		2011–2018	2.9 (−1.2; 7.3)	

* *p* < 0,05.

## Data Availability

The data presented in this study are available on request from the corresponding author.
